# Mortality Prediction by Kinetic Parameters of Lactate and S-Adenosylhomocysteine in a Cohort of Critically Ill Patients

**DOI:** 10.3390/ijms25126391

**Published:** 2024-06-09

**Authors:** Jochen J. Schoettler, Kathrin Brohm, Sonani Mindt, Evelyn Jäger, Bianka Hahn, Tanja Fuderer, Holger A. Lindner, Verena Schneider-Lindner, Joerg Krebs, Michael Neumaier, Manfred Thiel, Franz-Simon Centner

**Affiliations:** 1 Department of Anesthesiology, Surgical Intensive Care Medicine and Pain Medicine, Medical Faculty Mannheim, University Medical Center Mannheim, University of Heidelberg, Theodor-Kutzer-Ufer 1-3, 68167 Mannheim, Germany; jochen.schoettler@umm.de (J.J.S.); franz-simon.centner@umm.de (F.-S.C.); 2 Merck KGaA (SQ-Animal Affairs), Frankfurterstrasse 250, 64293 Darmstadt, Germany; 3 Institute for Clinical Chemistry, Medical Faculty Mannheim, University Medical Center Mannheim, University of Heidelberg, Theodor-Kutzer-Ufer 1-3, 68167 Mannheim, Germany; 4 Institute for Laboratory and Transfusion Medicine, Hospital Passau, Innstrasse 76, 94032 Passau, Germany

**Keywords:** organ dysfunction, lactate, s-adenosylhomocysteine, mortality prediction, critically ill patients

## Abstract

Tissue hypoxia is associated with the development of organ dysfunction and death in critically ill patients commonly captured using blood lactate. The kinetic parameters of serial lactate evaluations are superior at predicting mortality compared with single values. S-adenosylhomocysteine (SAH), which is also associated with hypoxia, was recently established as a useful predictor of septic organ dysfunction and death. We evaluated the performance of kinetic SAH parameters for mortality prediction compared with lactate parameters in a cohort of critically ill patients. For lactate and SAH, maxima and means as well as the normalized area scores were calculated for two periods: the first 24 h and the total study period of up to five days following ICU admission. Their performance in predicting in-hospital mortality were compared in 99 patients. All evaluated parameters of lactate and SAH were significantly higher in non-survivors compared with survivors. In univariate analysis, the predictive power for mortality of SAH was higher compared with lactate in all forms of application. Multivariable models containing SAH parameters demonstrated higher predictive values for mortality than models based on lactate parameters. The optimal models for mortality prediction incorporated both lactate and SAH parameters. Compared with lactate, SAH displayed stronger predictive power for mortality in static and dynamic application in critically ill patients.

## 1. Introduction

Tissue hypoxia is a crucial factor for the development of organ dysfunction in critically ill patients and is directly related to morbidity and mortality [1,2]. During tissue hypoxia, because of impaired mitochondrial oxidation, lactate is overproduced and underutilized, resulting in hyperlactatemia [3]. As tissue hypoxia increases blood lactate levels in animals [4] and humans [5,6], lactate measurements are clinically used to quantify hypoxia [3,7]. However, the causes for hyperlactatemia are multifactorial and reflect a complex metabolic disturbance in which increased aerobic and anaerobic production and decreased clearance are important elements [8,9,10]. Nevertheless, multiple studies showed that blood lactate levels relate to mortality in patients treated in the ICU, independently from the underlying critical illness [8,9,11,12,13]. However, the predictive power of a single lactate value is limited [14,15,16], and consecutive studies have revealed that serial evaluations of lactate and derived values strengthen the predictive value regarding mortality [17,18,19]. In this context, a calculated parameter incorporating the sum of the areas under the curve (AUCs) of serial lactate levels was introduced to capture the severity and duration of hyperlactatemia [19,20,21,22,23,24,25], which is normalized in most studies for the observed time interval [20,21,23,24,25]. This concept, termed “lactate area” [22], “lactate area score” [19,23,24], or “lactate load” [20,21,25], is supposed to reflect the hypoxic burden of a patient and was shown to be a superior predictor of 28-day mortality in patients with and without sepsis compared with other forms of lactate application for this purpose [19,20,21]. 

S-adenosylhomocysteine (SAH) is another metabolite that reflects tissue hypoxia [26,27]. Recently, it was shown that SAH allowed for improved prediction of septic organ dysfunction and death compared with lactate when using a single value close to ICU admission [28]. To the best of our knowledge, the kinetic parameters of SAH like mean, maximum, and normalized area score have not been evaluated for mortality prediction before. Therefore, in this study, we aimed to investigate the predictive power of kinetic parameters of SAH for mortality compared with those of lactate. For every evaluated parameter, namely the initial value, mean, maximum, and normalized area score, SAH had stronger predictive power for mortality compared with lactate. Multivariable models containing SAH parameters had higher predictive values for death than models with lactate parameters. The optimal models for mortality prediction incorporated both lactate and SAH parameters.

## 2. Results

### 2.1. Baseline and Clinical Characteristics of Study Participants

A total of 99 patients with an in-hospital mortality of 24% were analyzed. Survivors and non-survivors significantly differed in age, primary diagnosis of polytrauma, vascular comorbidity, length of stay in the ICU, as well as initial values of creatinine, urea, potassium, Simplified Acute Physiology Score (SAPS) II, Sequential Organ Failure Assessment (SOFA) Score, lactate, and SAH (Table 1).

In the univariate logistic regression analysis for the prediction of death, the statistically significant univariate variables at baseline in descending order were SAPS II, SAH, urea, creatinine, vascular comorbidity, SOFA, acidosis, age, potassium, base excess, and standard bicarbonate (Supplementary Table S1). Notably, initial lactate did not reach the level of statistical significance for the prediction of death in the univariate logistic regression analysis (*p* = 0.066).

### 2.2. Time Course of Lactate and SAH Plasma Concentrations in Relation to Mortality

**Figure 1:**
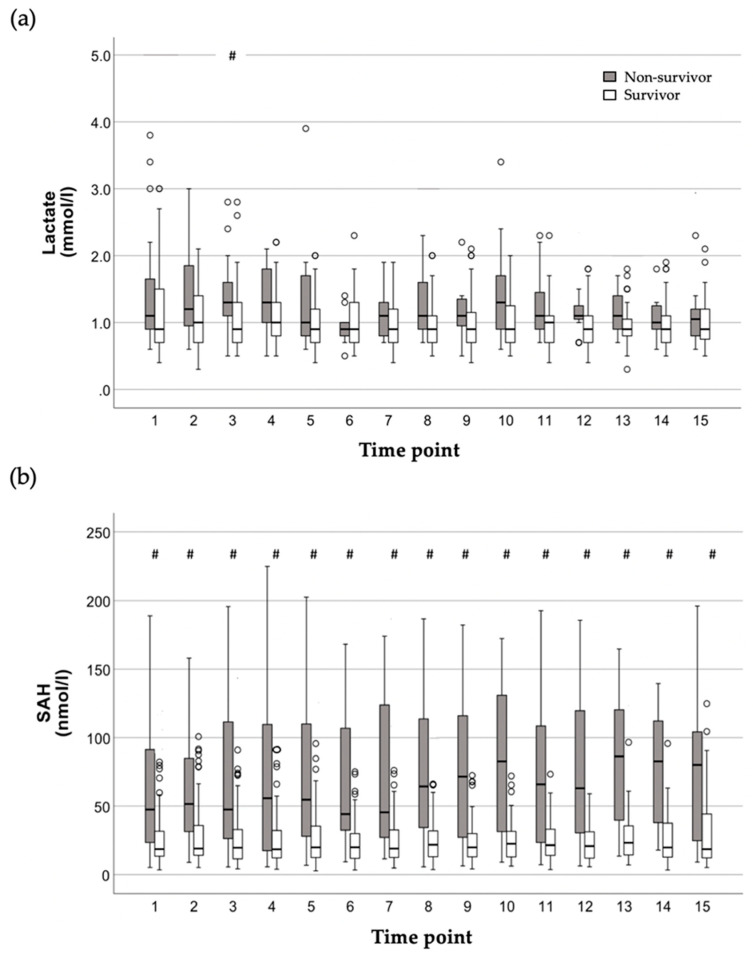
Time course of lactate (**a**) and S-adenosylhomocysteine (SAH) (**b**) plasma concentrations presented for each of up to 15 evaluation time points within the study period spanning up to five days (= 112 h), grouped by in-hospital mortality. Data of non-survivors and survivors are shown by filled and empty box plots, with the upper and lower lines of the box representing the 25th and 75th percentiles, respectively. The horizontal line in the box represents the median value. Empty circles indicate outliers higher than 1.5-fold but less than 2.5-fold of the interquartile range. The data were compared between non-survivors and survivors at each time point within the study period spanning up to five days and 15 blood samplings using the Mann–Whitney U test, and levels of significance were corrected according to Bonferroni [29]. # indicates statistically significant differences between non-survivors and survivors. Except for time point 3, lactate plasma concentrations (**a**) were not significantly different between non-survivors and survivors throughout the study period. In contrast, SAH plasma concentrations (**b**) were significantly higher in non-survivors compared with survivors at every time point within the study period.

Plasma concentrations of lactate and SAH were compared between non-survivors and survivors at each time point within the study period spanning up to five days, and the levels of significance were corrected according to Bonferroni [29]. Except for time point 3, lactate plasma concentrations were not significantly different between non-survivors and survivors (Figure 1a). In contrast, SAH plasma concentrations were significantly higher in non-survivors compared with survivors at every time point within the study period (Figure 1b).

### 2.3. Correlations of Kinetic Parameters of Serially Determined Lactate and SAH Plasma Concentrations with Each Other as Well as Clinical Scores

For the first 24 h following study inclusion, the Spearman coefficients for the correlation between the corresponding lactate parameters and SAH parameters ranged from 0.42 to 0.45 (Table 2, values to the right above the diagonal line of auto-correlations). Compared with lactate parameters, SAH parameters showed stronger correlations with the prognostic clinical scores for critically ill patients, namely SAPS II and SOFA: While the correlation between SAPS II and lactate parameters ranged between 0.25 and 0.33, it was between 0.50 and 0.52 for SAH parameters. The correlation between SOFA and lactate parameters was between 0.28 and 0.34, while it ranged from 0.37 to 0.38 for SAH parameters (Table 2).

During the total study period, the correlation between corresponding lactate and SAH parameters was slightly weaker compared with the early period (0.36–0.44 vs. 0.42–0.45, Table 2, values to the left under the diagonal line of auto-correlations). Although the correlations between lactate parameters and clinical scores tended to strengthen over the total study period, they remained weaker than those of SAH parameters in all applications. Notably, the correlations between mean SAH and normalized SAH area score with SAPS II were slightly weaker for the total study period than for the early period, while their correlation with SOFA strengthened (Table 2).

### 2.4. Kinetic Parameters of Serially Determined Lactate and SAH Plasma Concentrations and Their Relation to Mortality

For both periods, all parameters of lactate and SAH differed significantly between survivors and non-survivors (Table 3). However, although statistically significant, the initial, maximum, mean, and normalized area score values for lactate showed only slight differences of 0.2 to 0.5 mmol/L between survivors and non-survivors and were all below an absolute value of 2 mmol/L. In contrast, for SAH, the values of non-survivors were between 2.3 and 2.6 times higher as in survivors. The statistical significance in the case of lactate and the fold difference in the case of SAH were stronger when the total study period was considered for the analyses (Table 3).

**Figure 2:**
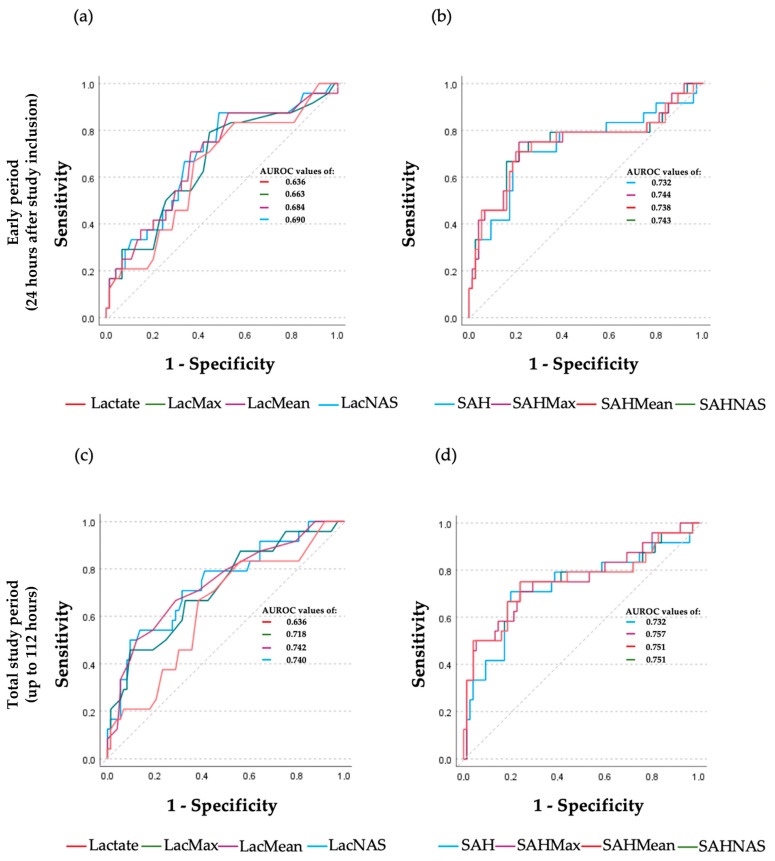
Area under the receiver operating characteristic (AUROC) analysis for in-hospital mortality of kinetic parameters of lactate and S-adenosylhomocysteine (SAH), calculated for the first 24 h following study inclusion (**a**,**b**) and the total study period spanning up to 5 days (= 112 h; (**c**,**d**)). Panel (**a**): ROC curves calculated for initial (Lactate), maximum (LacMax), and mean (LacMean) lactate as well as normalized lactate area score (LacNAS) within the early observation period. Panel (**b**): ROC curves calculated for initial (SAH), maximum (SAHMax), and mean (SAHMean) SAH as well as normalized SAH area score (SAHNAS) within the early observation period. There were no statistically significant differences within and between group comparisons of the AUROCs of lactate and SAH parameters, respectively. Panel (**c**): ROC curves of lactate parameters, calculated for the total study period. AUROCs of the mean lactate and normalized lactate area score were significantly higher compared with initial lactate (*p* = 0.041 and *p* = 0.049, respectively). Panel (**d**): ROC curves of SAH parameters, calculated for the total study period. There were no statistically significant differences between the AUROCs of SAH parameters or between respective AUROCs of lactate and SAH parameters.

In the univariate logistic regression analysis, initial lactate had an AUROC of 0.636 for mortality and thereby had poor predictive power according to the classification by Hosmer and Lemeshow [30] (Table 4 and Figure 2a). However, initial SAH demonstrated an acceptable predictive value with an AUROC of 0.732. During the early period, all analyzed kinetic lactate parameters showed poor predictive power for mortality. In contrast, kinetic SAH parameters had acceptable predictive values (Table 4 and Figure 2b). However, the observed differences between the AUROCs of lactate and SAH parameters did not reach statistical significance.

When considering the total study period, all kinetic parameters gained predictive power for mortality, with AUROC values exceeding 0.7. When compared with the initial value, the AUROCs for the mean and normalized area score of lactate were significantly greater (*p* = 0.041 and *p* = 0.049), while the AUROCs for the SAH kinetic parameters did not differ significantly. Overall, SAH consistently outperformed lactate in terms of AUROC values, although statistical significance was not reached. Maximum SAH had the highest AUROCs for both periods, with values of 0.744 and 0.757, respectively (Table 4 and Figure 2).

Using the stepwise backward method, separate models for all investigated lactate and SAH parameters were examined in multivariable logistic regression analyses for the endpoint of mortality. The covariates included parameters that were significantly associated with mortality in the univariate analysis (Supplementary Table S1) but were not redundant with the variables contained in SAPS II.

The final models for in-hospital mortality are summarized in Table 5. In the models that included kinetic parameters calculated for the first 24 h following study inclusion, the AUROCs for the initial, mean, and maximum values were between 0.781 and 0.788 for lactate, while they ranged between 0.802 and 0.810 for SAH. For the normalized area score, both lactate and SAH had AUROCs of 0.805 (Table 5).

When kinetic parameters calculated for the total study period were incorporated into the multivariable models, the AUROCs gained strength for predicting mortality, with values ranging between 0.830 and 0.842. Although the AUROC values for kinetic parameters of SAH were slightly greater than those for lactate, this difference did not reach statistical significance (Table 5).

To further compare the contribution of each parameter to mortality prediction, the combination of covariates of the final multivariable models for SAH parameters shown in Table 5 were fixed, and the respective SAH parameter was replaced by the corresponding lactate parameter. Meanwhile, for the period spanning the first 24 h following study inclusion, the AUROC values for all SAH parameters were above 0.8; they remained below 0.8 for all corresponding lactate parameters in this analysis (Supplementary Table S2). When the total study period was included in this modeling approach, the AUROC values for the SAH parameters were consistently higher than those of the corresponding lactate-parameters, although the differences were not statistically significant (Supplementary Table S2).

With the aim of determining an optimal multivariable model for mortality prediction, a final analysis step employed the stepwise backward method using the same covariates as above (Table 5). However, this time, instead of entering lactate and SAH parameters separately into the modeling process, they were included together as a combined set of variables. For the 24 h period following study inclusion, the final model included the following variables with an AUROC of 0.840: SAPS II, maximum SAH, lactate, and lactate area score. When considering the total study period, the final model achieved an AUROC of 0.869, and the relevant variables were SAPS II, mean SAH, lactate, and maximum lactate (Table 6).

## 3. Discussion

The development of organ dysfunction has a major impact on the clinical course of critically ill patients and directly relates to morbidity and mortality [1,2]. Tissue hypoxia is a crucial factor within the pathophysiological events leading to organ dysfunction and death [1,2]. Although the pathophysiology underlying hyperlactatemia is complex, incorporating anaerobic and aerobic disturbances [8,10], lactate is the marker primarily used in clinical practice to detect and quantify tissue hypoxia in critically ill patients [3,7,31]. Lactate adds to risk-stratification for mortality in a variety of conditions in critical care [8] and is considered the main prognostic parameter of mortality in patients with shock [10,19,32]. Consequently, the measurement of lactate was introduced into the 1 h bundle of the Surviving Sepsis Campaign (SSC) guidelines [31].

Although increased lactate levels at ICU admission have prognostic value for mortality, independently from the underlying critical illness, the predictive power is limited [7,8,9,11,33]. Serial evaluations of lactate and derived values strengthen the predictive power regarding mortality [17,18,19,20]. In consecutive studies, the form of lactate application for mortality prediction was refined, and the lactate area score as the sum of the AUC of serial lactate levels was introduced as a concept capturing the severity and duration of hyperlactatemia [19,20]. Accordingly, the lactate area score was confirmed as a superior prognostic marker for the prediction of mortality in patients with septic shock compared with other forms of lactate application [19]. Thus, we chose to investigate the normalized lactate area score in the present study because it is supposed to capture the hypoxic burden of a patient by determining the severity and duration of hyperlactatemia [19,20,22]. Lactate clearance is another approach to evaluate lactate and its prognostic value, but in contrast to the lactate area score, it cannot capture the concepts of severity and burden of hypoxia [19,22]. 

In our cohort of critically ill patients, the initial lactate and all kinetic lactate parameters calculated for the first 24 h following study inclusion had AUROCs < 0.7, thereby having poor predictive power [30] for in-hospital mortality in the univariate analysis, which was similarly observed by Chen et al. in patients with septic shock in the MIMIC III database [20]. Normalized lactate area score and mean lactate had the best predictive values with AUROCs of 0.684 and 0.690 for the early period of the first 24 h following study inclusion. In a study by Yu et al., the AUROCs for mortality of the early normalized lactate area score was 0.66 compared with 0.60 and 0.64 for initial and maximum lactate in patients with septic shock [24]. In geriatric patients with septic shock, Wang et al. observed an AUROC of 0.76 for the early normalized lactate area score compared with 0.63 and 0.67 for initial lactate and lactate clearance, respectively [23]. In a mixed cohort of critically ill patients analyzed by Chen et al. again, the early normalized lactate area score had the strongest predictive power for mortality (AUROC 0.71) compared with the maximum or mean lactate (AUROCs of 0.68 and 0.69), respectively [21]. Taken together, although the form of application of lactate was improved, the predictive power of the early lactate area score for mortality is still not very strong [21]. This might be due to the circumstance where, although lactate is often used as a marker for hypoxia, it is not ideal for this purpose due to a lack of specificity. Other pathophysiologic features than hypoxia can lead to an accumulation of lactate, such as the acceleration of aerobic glycolysis via adrenergic stimulation [34], the inhibition of mitochondrial pyruvate dehydrogenase [35], or hampered hepatic metabolization [36]. Thus, increased lactate levels reflect a complex metabolic disturbance contributed to by increased aerobic and anaerobic production and decreased clearance [9].

In the search for an alternative marker for hypoxia in critically ill patients, in addition to its association with an elevated risk of cardiovascular disease [37,38] and renal disease in diabetic nephropathy [39] and chronic kidney disease [40], SAH has also emerged as a metabolite reflecting tissue hypoxia [26,27,28,41]. Hypoxia causes the breakdown of energy-rich adenine nucleotides, leading to an intracellular accumulation of adenosine [27]. Adenosine reacts with L-homocysteine to form stable SAH [42]. Thus, SAH is considered to be a marker for a lack of perfusion-dependent oxygen supply and hence tissue hypoxia [26,27,28,41]. SAH was found to be a highly sensitive marker for myocardial ischemia [26]. Furthermore, in ischemic renal tissue, a long-lasting increase in SAH was observed, even after restoration of perfusion [41]. SAH was shown to be associated with acute kidney injury in critically ill patients [43]. Wexler et al. were the first to observe increased SAH plasma concentrations in non-survivors compared with survivors in a cohort of septic patients [44]. Recently, SAH was shown to have higher predictive power for septic organ dysfunction and death compared with lactate when a single value close to ICU admission was analyzed [28]. Interestingly, in the context of exploring vascular senescence, a correlation between elevated SAH plasma levels and mitochondrial dysfunction was discussed [38], with the latter also playing a crucial role in the development of organ dysfunction during critical illness [45].

To the best of our knowledge, this is the first study evaluating kinetic parameters of SAH for mortality prediction in critically ill patients. Because of the above-discussed status of lactate as an established marker for hypoxia and mortality prediction in critically ill patients, we chose lactate as a reference.

Although lactate and SAH are both supposed to capture hypoxia, the correlation between corresponding parameters of lactate and those of SAH was fair, with the correlation coefficients ranging from 0.42 to 0.45 [46], which might be due to the multifactorial etiology of hyperlactatemia discussed above. Of note, the correlation with SAPS II and SOFA as established prognostic scores for critically ill patients was higher for SAH parameters than for lactate parameters.

In our study, although statistically significant, the medians of initial, maximum, and mean values of lactate showed only slight differences, ranging from 0.2 to 0.5 mmol/l between survivors and non-survivors. Regarding these small differences between survivors and non-survivors, these values are not very useful in clinical practice for prognostication on an individual level. Furthermore, with an absolute value below 2 mmol/l, the medians of initial, mean, and maximum lactate were within the range of values regarded as normal [3] even in non-survivors, thereby missing the threshold for clinical intervention [31,47]. In contrast, SAH values in non-survivors were between 2.3 and 3 times higher than in survivors, making it potentially more useful for clinical practice than lactate. Furthermore, while lactate was significantly different between non-survivors and survivors at only one time point throughout the study period, SAH was significantly higher in non-survivors compared with survivors at every time point within the study period, which was robust after adjustment for multiple testing. Although missing statistical significance, for every evaluated parameter, namely initial value, mean, maximum, and normalized area score, SAH had greater AUROC values for mortality compared with lactate in univariate analyses. Additionally, all multivariable models containing SAH parameters had greater AUROCs compared with those including lactate parameters, except for the early normalized area score with identical AUROC values. Taken together, the results of the present study strongly suggest that in critically ill patients, kinetic parameters of SAH might be more useful for mortality prediction than those of lactate. Nevertheless, the best predictive performance was achieved when the parameters of lactate and SAH were combined in multivariable models, suggesting a joint effect for mortality prediction.

A further aspect that might favor the practicability of SAH as a mortality predictor compared with lactate is the observation time needed to reach an acceptable predictive value: most studies using the lactate area score used a 24 h period following ICU admission or onset of shock, respectively, and therefore, the parameter was called “early lactate area”. Due to our comprehensive study design, we were able to extend the period to up to 5 days. Our rationale for performing this was that in critically ill patients, there is evidence for a relationship between the duration and severity of hyperlactatemia and mortality [19,48], an association between mortality and delayed lactate clearance [17], and a better mortality prediction of lactate values after 24 h following ICU admission [49]. Furthermore, the accuracy of the lactate area score was improved when more lactate measurements were obtained in non-sepsis patients [21], and it was suggested that the prognostic value of lactate area scores may increase with time [24]. Indeed, for lactate parameters, the extension of the observation period from 24 h to up to 112 h resulted in a better predictive performance, although not reaching the level of statistical significance. The poor predictive value of all evaluated lactate parameters within the first 24 h following study inclusion improved to acceptable AUROC values by prolonging the observed period. In the case of SAH, mortality prediction was already acceptable within the first 24 h following study inclusion and improved marginally when using a longer observation period. Additionally, with the application of a longer observation period, multivariable models gained relatively more predictive value for lactate compared with SAH parameters. Taken together, the comparison of the mortality association of the kinetic parameters between the period of 24 h versus 112 h suggests a more stable relation of SAH parameters with mortality than is the case for lactate parameters. In conclusion, SAH parameters seem to be of greater value for early mortality prediction than lactate parameters in critically ill patients.

### Study Limitations 

This was a single-center study that included a limited number of patients due to the laborious study design. Despite the limited number of study patients, the main results regarding SAH and lactate reached acceptable confidence intervals. Our study was limited to surgical ICU patients and patients with severe respiratory dysfunction due to the specialization of our center, which poses the risk of impaired generalizability. Nevertheless, many studies investigating the lactate area score focused analysis on patients suffering from one condition, making our approach more general. The concept of area score cannot capture kinetic changes within the analyzed period but is instead designed to reflect a burden of hypoxia.

## 4. Materials and Methods

### 4.1. Ethics

This was a secondary analysis of data obtained in a monocentric, prospective observational clinical study [28], which was approved by the local ethics committee (see also the Institutional Review Board Statement) and registered in the German Clinical Trials Register (DRKS00029970). All patients or their legal representatives gave informed consent. After recovery, previously non-self-consenting patients had the opportunity to withdraw their consent to participate in this study. Study design and reporting were based on the recommendations of the STROBE statement (https://www.strobe-statement.org, accessed 24 April 2024). The study was conducted in the 24-bed intensive care unit (ICU) of the Department of Anesthesiology, Surgical Intensive Care Medicine and Pain Medicine, Mannheim University Hospital, Medical Faculty Mannheim, University of Heidelberg, from June 2017 to June 2019.

### 4.2. Study Population

Due to the original study design, critically ill patients with either a continuous SIRS status [50] for two consecutive days or patients fulfilling sepsis criteria of the sepsis-1/2 definition but not severe sepsis or septic shock [50] within the first 24 h after ICU admission were included. Exclusion criteria were age < 18 years, immunosuppression, end-stage renal failure, pregnancy, ECMO therapy, and neurosurgical main diagnosis to exclude confounding neuroinflammation.

### 4.3. Collection of Data and Blood Sampling

The clinical data of ICU patients were collected via the Philips Intelli Space Critical Care and Anesthesia (ICCA)™ system. The initial blood samples were taken at study inclusion (=at baseline) and every 8 h for up to 15 time points over the consecutive five days, provided patients stayed in the ICU. For SAH measurements, venous blood samples were taken via a central catheter or a peripheral indwelling cannula. The laboratory staff had no information about the patients’ condition; likewise, the medical specialists were blinded to the SAH measurements. Lactate was determined via arterial blood gas analyses using a blood gas analyzer (Radiometer ABL 800 Flex, Radiometer, Willich, Germany).

### 4.4. Determination of Plasma Concentrations of SAH

SAH plasma concentrations were determined by stable-isotope dilution LC–tandem MS/MS following the separation of analytes by pH-dependent solid-phase extraction columns containing phenylboronic acid as previously described [51]. In our protocol, intra- and inter-assay CVs were around 4% and 8%, and the mean recoveries were 85%. The lower limit of detection was 1 nmol/l.

### 4.5. Calculation of Kinetic Parameters

For the calculation of kinetic parameters (maximum, mean, and normalized area score) of lactate and SAH, two periods were analyzed:

1. Early period: The 24 h following study inclusion, containing four measurements (time points 1 to 4) of lactate and SAH per patient, respectively.
2. Total study period: Up to 15 measurements of lactate and SAH per patient over a span of up to five days (=112 h). Altogether, 1126 lactate and 1248 SAH measurements were analyzed for this period.

The means and maxima of lactate and SAH plasma concentrations were calculated for these two periods. The area score is represented by the sum of the AUCs over time, following the approach by Chen et al. [20], to capture the cumulative effect of the respective metabolite over time. To represent the average intensity and ensure a normalization for the observed interval of time, as in most comparable studies, the lactate area score was divided by the respective time period to calculate the so-called normalized lactate area score [20,21,23,24,25]. Furthermore, in most studies using the concept of area scores, the observed period was restricted to the first 24 h following admission, and the resulting value was called the “early lactate area score”. Therefore, the investigation of the “early” period in this study ensured comparability of our results and additionally allowed us to investigate the effect of different periods on the significance of the respective kinetic parameters.

### 4.6. Statistical Analyses

For clinical characterization, continuous variables were compared by a *t*-test (Satterthwaite method), and categorical variables were assessed using the chi^2^ test. Non-normally distributed variables were compared using the Mann–Whitney U test. All variables are presented as medians (interquartile ranges (IQRs)) unless otherwise indicated. Categorical variables are expressed as numbers and percentages. For the analyses of correlations between the parameters of lactate and SAH as well as clinical scores, Spearman’s rank correlation coefficients were calculated. The univariate logistic regression analysis was performed to test for associations between parameters of lactate and SAH, respectively, and in-hospital mortality. The performance of univariate logistic regression models was evaluated by the estimated coefficients and calculated odds ratios. The estimated coefficient used as an exponent to the basis of 27,182 represents the factor by which the odds ratio of in-hospital death changes when the variable of interest increases by 1 unit. The predictive power of a variable of interest, i.e., the ability of the variable to discriminate between patients who survive and patients who die, was derived by the area under the receiver operating characteristics (AUROCs) curve. The differences of AUROCs within and between groups were calculated and tested for statistical significance. Multiple logistic regression analysis was performed to identify the independent covariables (risk factors) for in-hospital death in separate models for the kinetic parameters calculated for the first 24 h following study inclusion and for the total study period. The covariables were pre-specified based on *p*-values below 0.05 as obtained from the univariate logistic regression (Supplementary Table S1). Statistically significant variables in univariate testing were considered in combination with SAPS II (as established score of prognosis in ICU patients [52] and the most significant variable for in-hospital death in univariate analysis) if the variables were not related to the calculation of SAPS II to avoid redundancy. The listed covariables were sequentially removed if comparisons between the combined model and the base model were not statistically significant (likelihood ratio test *p*-value > 0.10), starting with the covariable least strongly associated with the outcome variable (backward stepwise method). This was continued until the association with the in-hospital death of all covariables remaining in the model was statistically significant. AUROCs were derived for the final models. The evaluation of the performance of AUROCs followed the suggestions by Hosmer and Lemeshow [30]. No outlier or extreme value corrections and no imputation of missing data points were performed in any case of directly measured or calculated variables. Despite the prediction of results according to hypotheses, all tests in this study were two-sided, and a *p*-value of less than 0.05 was considered to be statistically significant. In the case of multiple comparisons, the levels of significance were corrected according to Bonferroni [29]. Statistical analyses were performed using SAS Software V9.4 (SAS Institute, Cary, NC, USA) or IBM SPSS Statistics version 27 (IBM, Albany, NY, USA).

## Figures and Tables

**Table 1:** Baseline and clinical characteristics of study participants.

		All (N = 99)	Survivors (S) (N = 75)		Non-Survivors (NS) (N = 24)		
	n		n		n		S vs. NS
**Demographics**							
Age (years)	99	63 (53–76)	75	61 (49–74)	24	73 (60–79)	**0.002**
Male (%)		65 (66)		46 (61)		19 (79)	0.109
BMI [kg/m^2^]		26.1 (24.2–29.4)		25.8 (24–29.3)		26.7 (24.6–30.6)	0.246
**Primary diagnosis (%)**							
Major surgery		11 (11)		8 (11)		3 (13)	0.725
Sepsis		20 (20)		12 (16)		8 (33)	0.082
Cardiac arrest		2 (2)		2 (3)			
Polytrauma		42 (42)		36 (48)		6 (25)	**0.047**
Major bleeding		14 (14)		9 (12)		5 (21)	0.318
Resp. insuff./ARDS		10 (10)		8 (11)		2 (8)	1.000
**Comorbidities (%)**							
Cardiac		35 (35)		25 (33)		10 (42)	0.457
Vascular ^1^		21 (21)		11 (15)		10 (42)	**0.005**
Arterial hypertension		51 (52)		36 (48)		15 (63)	0.216
Pulmonary		12 (12)		8 (11)		4 (17)	0.478
Renal		20 (20)		12 (16)		8 (33)	0.082
Hepatic		6 (6)		4 (5)		2 (8)	0.630
Diabetes mellitus		17 (17)		13 (17)		4 (17)	1.000
Metabolic		10 (10)		8 (11)		2 (8)	1.000
Cerebral		11 (11)		9 (12)		2 (8)	1.000
Smoking		7 (7)		4 (5)		3 (13)	0.355
Alcoholism		6 (6)		3 (4)		3 (13)	0.151
**Clinical course**							
Mechanical ventilation (%)		86 (86.9)		64 (85.3)		22 (91.7)	0.501
Vasopressor therapy (%)		68 (69)		48 (64)		20 (83)	0.088
Volume balance (mL) ^2^	97	22.8 (−106.6–128.0)	74	31.5 (−83.6–130.1)	23	−12.4 (−162.4–129.2)	0.309
ICU-LOS (days)	99	25 (16–47)	75	27 (18–53)	24	19 (13–34)	**0.028**
In-hospital mortality (%)		24 (24)					
**Clinical chemistry at baseline**							
Creatinine (mg/dL)	96	0.98 (0.73–1.50)	72	0.88 (0.68–1.26)	24	1.72 (1.05–2.72)	**0.003**
Urea (mg/dL)	96	45.1 (33.4–63.8)	72	42.2 (30.8–55.8)	24	60.1 (43.7–81.7)	**0.007**
K^+^ (mmol/L)	98	4.1 (3.8–4.3)	74	4.0 (3.8–4.2)	24	4.3 (4.1–4.5)	**0.041**
Bilirubin (mg/dL)	93	0.61 (0.35–0.94)	71	0.61 (0.34–0.97)	22	0.625 (0.41–0.94)	0.552
AST (U/L)	90	43 (27–90)	69	42 (29–93)	21	48 (26–78)	0.854
ALT (U/L)	92	36.5 (20–85)	70	39 (20–88)	22	28 (20–82)	0.857
Lipase	90	85.5 (61–207)	69	87 (61–193)	21	82 (61–357)	0.854
CRP (mg/dL)	96	150 (90–218)	72	152 (91–220)	24	146 (86–216	0.460
PCT (ug/L)	69	0.63 (0.20–2.29)	52	0.585 (0.185–2.125)	17	0.9 (0.32–2.29)	0.293
**Hematology at baseline**							
Hb (g/dL)	98	8.85 (8.10–10.1)	74	8.85 (7.9–10.1)	24	8.85 (8.4–9.65)	0.880
WBC (10^9^/L)	96	11.5 (8.29–14.3)	72	11.87 (9.01–14.31)	24	10.43 (5.93–14.31)	0.405
Thrombocytes (10^9^/L)	96	162 (106–250)	72	166 (114–256)	24	137 (94.5–244.5)	0.875
INR	96	1.07 (1–1.12)	72	1.055 (1–1.11)	24	1.1 (1–1.19)	0.589
**Vital signs at baseline**							
Temperature (°C)	96	37.1 (36.8–37.7)	72	37.1 (36.8–37.7)	24	37.1 (36.8–37.6)	0.993
Resp. rate (1/min)	98	19 (16–22)	74	19 (16–22)	24	20 (16–25)	0.199
Horovitz (mmHg)	98	280 (220–343)	74	301 (229–347)	24	218 (185–297)	0.102
Shock index	98	0.70 (0.61–0.88)	74	0.71 (0.60–0.87)	24	0.69 (0.62–0.95)	0.491
**Clinical scores at baseline**							
RASS	97	−3 (−5–0)	74	−3 (−5–−1)	23	−1 (−5–0)	0.298
TISS	98	18 (10–22)	74	17.5 (10–22)	24	22 (11.5–25)	0.092
SAPS II	98	35 (28–43)	74	33 (25–39)	24	42 (36–51)	**<0.001**
SOFA	99	8 (5–11)	75	8 (5–10)	24	11 (7–13)	**0.013**
**Hypoxia biomarkers at baseline**							
Lactate (mmol/L)	98	1 (0.7–1.6)	74	0.9 (0.7–1.5)	24	1.1 (0.9–1.65)	**0.045**
SAH (nmol/L)	99	21.7 (13.5–47.8)	75	18.61 (13.86–32.01)	24	47.56 (23.43–91.32)	**<0.001**

Results are given as the median (interquartile range), number, or percentage (%). Significant results are highlighted in bold. ^1^ The variable “vascular comorbidity” included peripheral arterial occlusive disease, previous occlusions, or aneurysm formations of abdominal vessels, pulmonary artery embolisms, or strokes. ^2^ Within the first 24 h. Abbreviations: N = number of patients included; n = number of determinations for the variable; BMI = body mass index; Resp. insuff. = respiratory insufficiency; ARDS = acute respiratory distress syndrome; ICU = intensive care unit; LOS = length of stay; K^+^ = potassium AST = aspartate aminotransferase; ALT = alanine aminotransferase; CRP = C-reactive protein; PCT = procalcitonin; Hb = hemoglobin; WBC = white blood cell; INR = international normalized ratio; resp = respiratory; Horovitz = Horovitz index; RASS = Richmond Agitation and Sedation Score; TISS = Therapeutic Intervention Severity Score; SAPS = Simplified Acute Physiology Score; SOFA = Sequential Organ Failure Assessment Score.

**Table 2:** Correlations of initial values and kinetic parameters of serially determined lactate and SAH plasma concentrations with each other as well as clinical scores.

		Early Period (24 h Following Study Inclusion, Time Points 1–4)									
		Lactate	LacMax	LacMean	LacNAS	SAH	SAHMax	SAHMean	SAHNAS	SAPS	SOFA
**Total study period (up to 112 h, time points 1–15)**	**Lactate**	1.00	0.85 <0.01	0.84 <0.01	0.84 <0.01	0.44 <0.01	0.44 <0.01	0.46 <0.01	0.45 <0.01	0.33 <0.01	0.34 <0.01
	**LacMax**	0.75 <0.01	1.00	0.90 <0.01	0.92 <0.01	0.41 <0.01	0.43 <0.01	0.44 <0.01	0.44 <0.01	0.25 <0.01	0.30 <0.01
	**LacMean**	0.73 <0.01	0.92 <0.01	1.00	0.92 <0.01	0.43 <0.01	0.44 <0.01	0.45 <0.01	0.45 <0.01	0.31 <0.01	0.28 0.01
	**LacNAS**	0.70 <0.01	0.90 <0.01	0.99 <0.01	1.00	0.40 <0.01	0.42 <0.01	0.43 <0.01	0.42 <0.01	0.28 0.01	0.33 <0.01
	**SAH**	0.44 <0.01	0.28 0.01	0.32 <0.01	0.34 <0.01	1.00	0.93 <0.01	0.94 <0.01	0.93 <0.01	0.51 <0.01	0.37 <0.01
	**SAHMax**	0.40 <0.01	0.40 <0.01	0.41 <0.01	0.43 <0.01	0.82 <0.01	1.00	0.99 <0.01	0.98 <0.01	0.50 <0.01	0.37 <0.01
	**SAHMean**	0.40 <0.01	0.34 <0.01	0.36 <0.01	0.38 <0.01	0.91 <0.01	0.94 <0.01	1.00	1.00 <0.01	0.51 <0.01	0.38 <0.01
	**SAHNAS**	0.40 <0.01	0.34 <0.01	0.36 <0.01	0.38 <0.01	0.90 <0.01	0.93 <0.01	1.00 <0.01	1.00	0.52 <0.01	0.38 <0.01
	**SAPS**	0.33 <0.01	0.25 0.02	0.34 <0.01	0.34 <0.01	0.51 <0.01	0.45 <0.01	0.49 <0.01	0.49 <0.01	1.00	0.10 0.34
	**SOFA**	0.34 <0.01	0.38 <0.01	0.34 <0.01	0.31 <0.01	0.37 <0.01	0.44 <0.01	0.41 <0.01	0.41 <0.01	0.10 0.34	1.00

Spearman’s correlation coefficients and corresponding *p*-values are shown. The initial value refers to the first time point of blood sampling following study inclusion. The maximum and mean give the highest or calculated averaged value of all collected values within the first 24 h following study inclusion and the total study period spanning up to 112 h, respectively. The normalized area scores were obtained by plotting the values of lactate and SAH for each patient within the respective period, followed by the division of the areas under the curves by the period of observation (for further explanation, see section “Calculation of kinetic parameters” within Materials and Methods). Abbreviations: SAH = S-adenosylhomocysteine; Lactate/SAH = initial value; Lac/SAHMax = maximum value within the respective period; Lac/SAHMean = mean value within the respective period; Lac/SAHNAS = normalized area score calculated for the respective period; SAPS = initial Simplified Acute Physiology Score; SOFA = initial Sequential Organ Failure Assessment Score.

**Table 3:** Initial values and kinetic parameters of serially determined lactate and SAH plasma concentrations within the first 24 h following study inclusion and the total study period, grouped by mortality.

		All (N = 99)	Survivors (S) (N = 75)	Non-Survivors (NS) (N = 24)	*p*-Value S vs. NS
**Early period** **(24 h following study inclusion)**	**Lactate (mmol/L)**				
	Initial/*n*	1.0 (0.7–1.6)/98	0.9 (0.7–1.5)/74	1.1 (0.9–1.7)/24	**0.045**
	Maximum/*n*	1.3 (1.0–1.8.0)/98	1.2 (0.9–1.7)/74	1.7 (1.3–2.3)/24	**0.016**
	Mean/*n*	1.1 (0.8–1.5)/371	1.0 (0.8–1.4)/278	1.3 (1.0–1.8)/93	**0.007**
	Normalized area score/*n*	1.0 (0.8–1.4)/371	0.7 (1.0–1.3)/278	1.2 (1.0–1.8)/93	**0.005**
	**SAH (nmol/L)**				
	Initial/*n*	21.7 (13.5–47.8)/99	18.6 (13.4–32.0)/75	47.6 (22.8–92.6)/24	**<0.001**
	Maximum/*n*	26.8 (17.5–52.6)/99	23.2 (17.4–40.8)/75	55.0 (32.6–122.3)/24	**<0.001**
	Mean/*n*	20.9 (14.2–42.8)/385	19.4 (14.1–32.3)/290	46.0 (25.1–98.6)/95	**<0.001**
	Normalized area score/*n*	21.6 (14.0–44.0)/385	19.9(13.8–33.1)/290	46.7 (26.0–100.1)/95	**<0.001**
**Total study period** **(up to 112 h)**	**Lactate (mmol/L)**				
	Maximum/*n*	1.4 (1.1–2.0)/98	1.4 (1.0–1.8)/74	1.9 (1.4–3.0)/24	**0.001**
	Mean/*n*	1.0 (0.8–1.3)/1126	0.9 (0.8–1.2)/859	1.4 (1.0–1.7)/267	**<0.001**
	Normalized area score/*n*	1.0 (0.8–1.3)/1126	1.0 (0.8–1.2)/859	1.4 (1.0–1.6)/267	**<0.001**
	**SAH (nmol/L)**				
	Maximum/*n*	33.7 (21.7–65.9)/99	30.4 (20.2–46.9)/75	88.5 (32.8–176.7)/24	**<0.001**
	Mean/*n*	23.3 (14.8–51.4)/1248	20.6 (14.2–29.2)/947	62.4 (24.8–122.6)/301	**<0.001**
	Normalized area score/*n*	23.9 (15.3–51.6)/1248	20.7(14.2–29.4)/947	62.9 (25.4–123.74)/301	**<0.001**

Results are given as the median (interquartile range). Significant results between survivors and non-survivors are highlighted in bold. Abbreviations: N = number of patients included; *n* = number of determinations for each parameter; SAH = S-adenosylhomocysteine.

**Table 4:** Univariate logistic regression analysis of initial values and kinetic parameters of serially determined lactate and SAH plasma concentrations within the first 24 h following study inclusion and the total study period for in-hospital mortality.

		Coefficient (Means, SE)	Odds Ratio (95% CI)	*p*-Value	AUROC (SE)	*p*-Value
**Early period** **(24 h following study inclusion)**	**Lactate**					
	Initial	0.446 (0.243)	1.563 (0.971–2.514)	0.066	0.636 (0.065)	**0.046**
	Maximum	0.587 (0.294)	1.816 (1.021–3.232)	**0.042**	0.663 (0.065)	**0.017**
	Mean	1.000 (0.442)	2.718 (1.142–6.469)	**0.024**	0.684 (0.064)	**0.007**
	Normalized area score	1.010 (0.432)	2.746 (1.177–6.407)	**0.019**	0.690 (0.062)	**0.005**
	**SAH**					
	Initial	0.028 (0.008)	1.028 (1.013–1.044)	**<0.001**	**0.732 (0.067)**	**0.001**
	Maximum	0.023 (0.006)	1.023 (1.011–1.035)	**<0.001**	**0.744 (0.068)**	**<0.001**
	Mean	0.027 (0.007)	1.027 (1.013–1.042)	**<0.001**	**0.738 (0.069)**	**<0.001**
	Normalized area score	0.026 (0.007)	1.027 (1.013–1.041)	**<0.001**	**0.743 (0.069)**	**<0.001**
**Total study period** **(up to 112 h)**	**Lactate**					
	Maximum	0.864 (0.305)	2.373 (1.306–4.313)	**0.005**	**0.718 (0.061)**	**0.001**
	Mean	2.452 (0.751)	11.607 (2.664–50.569)	**0.001**	0.742 (0.060) *	**0.001**
	Normalized area score	2.412 (0.753)	11.160 (2.552–48.800)	**0.001**	0.740 (0.060) *	**<0.001**
	**SAH**					
	Maximum	0.017 (0.005)	1.017 (1.008–1.027)	**<0.001**	**0.757 (0.064)**	**<0.001**
	Mean	0.030 (0.007)	1.030 (1.015–1.045)	**<0.001**	**0.751 (0.067)**	**<0.001**
	Normalized area score	0.026 (0.007)	1.027 (1.013–1.041)	**<0.001**	**0.751 (0.067)**	**<0.001**

Regression coefficients, odds ratios, and AUROCs are given as the mean, and the standard error (SE) or confidence interval (CI) is shown, respectively. *p*-values indicating significant results of univariate logistic regression analysis are highlighted in bold. While significant *p*-values of the AUROC analysis are given in bold, values of AUROCs are highlighted in bold only if both the respective logistic regression and AUROC analysis reached the level of statistical significance and the AUROC value was at least acceptable (AUROC ≥ 0,7) according to the classification by Hosmer and Lemeshow [30]. * indicates a significant (*p* < 0.05) increase compared with the initial value. There were no statistically significant differences between AUROCs of lactate and SAH parameters (*p*-values of comparisons: initial values: *p* = 0.171; early period: maxima: *p* = 0.325, means: *p* = 0.488, area scores: 0.517; total study period: maxima: *p* = 0.627, means: *p* = 0.896, area scores: 0.882). Abbreviations: AUROC = area under the receiver operator characteristic curve; SAH = S-adenosylhomocysteine.

**Table 5:** Multivariable logistic regression models for the prediction of mortality containing baseline parameters, initial values, and kinetic parameters of serially determined lactate or SAH plasma concentrations within the first 24 h following study inclusion and the total study period.

	Early Period (24 h Following Study Inclusion)				Total Study Period (Up to 112 h)			
	AUROC (*p*-Value)	Model Parameters	OR (95% CI)	*p*-Value of OR	AUROC (*p*-Value)	Model Parameters	OR (95% CI)	*p*-Value of OR
**Initial lactate**	0.781 **(<0.001)**	Vascular CM SAPS II	2.86 (0.93–8.85) 1.11 (1.04–1.17)	0.068 **0.001**	0.781 **(<0.001)**	Vascular CM SAPS II	2.86 (0.93–8.85) 1.11 (1.04–1.17)	0.068 **0.001**
**Maximum lactate**	0.788 **(<0.001)**	Vascular CM SAPS II Maximum lactate	2.81 (0.85–9.28) 1.10 (1.04–1.17) 1.60 (0.85–3.00)	0.097 **0.002** 0.111	0.830 **(<0.001)**	Vascular CM SAPS II Maximum lactate	2.87 (0.86–9.52) 1.08 (1.01–1.15) 2.29 (1.23–4.26)	0.086 **0.003** **0.008**
**Mean lactate**	0.781 **(0.001)**	SAPS II Mean lactate	1.10 (1.04–1.17) 2.46 (0.99–6.10)	**0.001** 0.053	0.838 **(<0.001)**	Vascular CM SAPS II Creatinine Mean lactate	2.94 (0.84–10.25) 1.06 (0.995–1.14) 1.71 (0.95–3.08) 8.45 (1.72–41.41)	0.091 0.072 0.076 **0.009**
**Normalized lactate** **area score**	0.805 **(<0.001)**	Vascular CM SAPS II Lactate area score	2.77 (0.85–8.98) 1.10 (1.04–1.17) 2.61 (1.08–6.34)	0.090 **0.002** **0.034**	0.837 **(<0.001)**	Vascular CM SAPS II Creatinine Lactate area score	2.91 (0.84–10.09) 1.06 (0.996–1.14) 1.67 (0.93–3.00) 7.85 (1.59–38.70)	0.092 0.067 0.085 **0.011**
**Initial SAH**	0.802 **(<0.001)**	SAPS II SAH	1.08 (1.01–1.15) 1.02 (1.002–1.04)	**0.023** **0.032**	0.802 **(<0.001)**	SAPS II SAH	1.08 (1.01–1.15) 1.02 (1.002–1.037)	**0.023** **0.032**
**Maximum SAH**	0.810 **(<0.001)**	SAPS II Maximum SAH	1.08 (1.02–1.15) 1.02 (1.01–1.03)	**0.013** **0.005**	0.836 **(<0.001)**	Vascular CM SAPS II Maximum SAH	2.88 (0.84–9.84) 1.09 (1.03–1.17) 1.01 (1.004–1.02)	0.091 **0.005** **0.004**
**Mean SAH**	0.804 **(<0.001)**	SAPS II Mean SAH	1.08 (1.01–1.15) 1.02 (1.01–1.04)	**0.018** **0.010**	0.842 **(<0.001)**	Vascular CM SAPS II Mean SAH	3.01 (0.86–10.59) 1.07 (1.01–1.14) 1.03 (1.01–1.06)	0.085 **0.034** **0.002**
**Normalized SAH** **area score**	0.805 **(<0.001)**	SAPS II SAH area score	1.08 (1.01–1.15) 1.02 (1.01–1.04)	**0.016** **0.009**	0.840 **(<0.001)**	Vascular CM SAPS II SAH area score	3.00 (0.87–10.38) 1.08 (1.02–1.15) 1.02 (1.01–1.04)	0.083 **0.015** **0.003**

Significant results are highlighted in bold. Abbreviations: AUROC = Area under the receiver operating characteristic curve; OR = odds ratio; CI = confidence interval; CM = comorbidity; SAPS = Simplified Acute Physiology Score; SAH = S-adenosylhomocysteine.

**Table 6:** Optimal multivariable logistic regression models for the prediction of death, containing baseline parameters and kinetic parameters of lactate and SAH.

	AUROC (*p*-Value)	Model Parameters	OR (95% CI)	*p*-Value of OR
**Early period** **(24 h following study inclusion)**	0.840	SAPS II	1.10 (1.03–1.17)	**0.007**
	**(<0.001)**	Maximum SAH	1.01 (1.00–1.03)	0.052
		Lactate	0.20 (0.04–1.12)	0.066
		Lactate area score	16.70 (1.41–197.78)	**0.026**
**Total study period** **(up to 112 h)**	0.869	SAPS II	1.08 (1.01–1.16)	**0.017**
	**(<0.001)**	Mean SAH	1.02 (1.01–1.04)	**0.007**
		Lactate	0.30 (0.09–0.989)	**0.048**
		Maximum lactate	5.01 (1.45–17.32)	**0.011**

Significant results are highlighted in bold. Abbreviations: AUROC = Area under the receiver operating characteristic curve; OR = odds ratio; CI = confidence interval; SAPS = Simplified Acute Physiology Score; SAH = S-adenosylhomocysteine.

## Data Availability

The datasets generated and analyzed during the current study are not publicly available due to patient privacy but are available from the corresponding author upon reasonable request.

## Supplementary Materials

The following supporting information can be downloaded at: https://www.mdpi.com/article/10.3390/ijms25126391/s1.
