# Supplementary material for: Mortality Prediction by Kinetic Parameters of Lactate and S-Adenosylhomocysteine in a Cohort of Critically Ill Patients
Source: Int J Mol Sci. 2024 Jun 9;25(12):6391. doi: 10.3390/ijms25126391 (PMC11204002; doi:10.3390/ijms25126391)
Supplement: Supplementary file 1 [file ijms-25-06391-s001.zip › Supplementary Table S1.pdf]

Supplementary Table S1. Univariate analysis for the prediction of in-hospital death.

Variable	p-value
SAPS II	**<0.001**
SAH (nmol/L)	**<0.001**
Urea (mg/dL)	**0.002**
Creatinine (mg/dL)	**0.005**
Comorbidity: vascular	**0.007**
SOFA	**0.008**
Acidosis (pH<7.35)	**0.010**
Age (years)	**0.013**
Potassium (mmol/L)	**0.026**
Standard base excess (mmol/L)	**0.029**
Standard bicarbonate (mmol/L)	**0.030**
Primary diagnosis: polytrauma	0.052
Horovitz Index [mmHg]	0.053
Lactate [mmol/L]	0.066
TISS	0.066
SpO2 [%]	0.071
Primary diagnosis: sepsis	0.072
Comorbidity: renal	0.072
Pressor therapy	0.096
Gender	0.116
Comorbidity: alcoholism	0.149
Respiratory rate [1/min]	0.152
Diastolic blood pressure [mmHg]	0.163
Chloride- [mmol/L]	0.167
PCO2 [mmHg]	0.172
Sodium+ [mmol/L]	0.173
Mean arterial pressure [mmHg]	0.190
Volume balance [mL]	0.190
Comorbidity: arterial hypertension	0.219
RASS	0.239
Comorbidity: smoking	0.247
Charlson Comorbidity Index	0.253
FiO2	0.271
Primary diagnosis: major bleeding	0.286
Eosinophils [%]	0.333
WBC [10E9/L]	0.347
Systolic blood pressure [mmHg]	0.375
ScvO2 [%]	0.391
Body Mass Index [kg/m^2]	0.398
Ionized Calcium++ [mmol/L]	0.419
Shock index	0.431
Comorbidity: pulmonary	0.436

Comorbidity: cardiac	0.458
Alkalosis (pH>7.45)	0.472
CRP [mg/L]	0.494
PCT [µg/L]	0.497
Mechanical ventilation	0.505
Basophils [%]	0.525
INR	0.545
Comorbidity: hepatic	0.595
Glucose [mg/dL]	0.611
Comorbidity: cerebral	0.621
SIDa [mmol/L]	0.628
Bilirubin [mg/dL]	0.670
Comorbidity: metabolic	0.742
Primary diagnosis: resp. Insuff. /ARDS	0.742
PO2 [mmHg]	0.762
Primary diagnosis: major surgery	0.804
Heart rate [1/min]	0.829
AST [U/L]	0.830
ALT [U/L]	0.835
Thrombocytes [10E9/L]	0.852
Neutrophils [%]	0.874
Lipase [U/L]	0.880
Hb [g/dL]	0.899
Comorbidity: diabetis mellitus	0.940
Monocytes [%]	0.972
Primary diagnosis: cardiac arrest	0.984
Temperature [°C]	0.993
Lymphocytes [%]	1.000

p-values in univariate testing for mortality prediction in descending order; Abbreviations: TISS=therapeutic intervention score scale; SpO2=peripheral capillary oxygen saturation; PCO2=partial pressure of carbon dioxide; RASS=Richmond agitation sedation scale; FiO2=fraction of inspired oxygen; WBC=white blood cell count; ScvO2=central venous oxygen saturation; CRP=c-reactive protein; PCT=procalcitonin; INR=international normalized ratio; SIDa=strong ion difference apparent; PO2=partial pressure of oxygen; AST=aspartate aminotransaminase; ALT=alanine aminotransaminase; Hb=hemoglobin.
