# Supplementary material for: Mortality Prediction by Kinetic Parameters of Lactate and S-Adenosylhomocysteine in a Cohort of Critically Ill Patients
Source: Int J Mol Sci. 2024 Jun 9;25(12):6391. doi: 10.3390/ijms25126391 (PMC11204002; doi:10.3390/ijms25126391)
Supplement: Supplementary file 1 [file ijms-25-06391-s001.zip › Supplementary Table S2.pdf]

Supplementary Table S2. Fixed multivariable logistic regression analyses for the prediction of mortality with replacement of SAH-parameters by corresponding lactate-parameters.

	**24 hours following study inclusion**				**Total study period (up to 112h)**			
	**AUROC (p-value)**	**Model parameters**	**OR (95% CI)**	**p-value of OR**	**AUROC (p-value)**	**Model parameters**	**OR (95% CI)**	**p-value of OR**
**Initial SAH**	0.802	SAPS II	1.08 (1.01-1.15)	**0.023**	0.802	SAPS II	1.08 (1.01-1.15)	**0.023**
	**(<0.001)**	SAH	1.02 (1.002-1.04)	**0.032**	**(<0.001)**	SAH	1.02 (1.002-1.037)	**0.032**
**Maximum SAH**	0.810	SAPS II	1.08 (1.02-1.15)	**0.013**	0.836	Vascular CM	2.88 (0.84-9.84)	0.091
	**(<0.001)**	Maximum SAH	1.02 (1.01-1.03)	**0.005**	**(<0.001)**	SAPS II	1.09 (1.03-1.17)	**0.005**
						Maximum SAH	1.01 (1.004-1.02)	**0.004**
**Mean SAH**	0.804	SAPS II	1.08 (1.01-1.15)	**0.018**	0.842	Vascular CM	3.01 (0.86-10.59)	0.085
	**(<0.001)**	Mean SAH	1.02 (1.01-1.04)	**0.010**	**(<0.001)**	SAPS II	1.07 (1.01-1.14)	**0.034**
						Mean SAH	1.03 (1.01-1.06)	**0.002**
**Normalized SAH area score**	0.805	SAPS II	1.08 (1.01-1.15)	**0.016**	0.840	Vascular CM	3.00 (0.87-10.38)	0.083
	**(<0.001)**	SAH area score	1.02 (1.01-1.04)	**0.009**	**(<0.001)**	SAPS II	1.08 (1.02-1.15)	**0.015**
						SAH area score	1.02 (1.01-1.04)	**0.003**
**Initial lactate**	0.776	SAPS II	1.11 (1.04-1.17)	**0.001**	0.781	Vascular CM	2.86 (0.93-8.85)	0.068
	**(<0.001)**	Lactate	1.37 (0.78-2.40)	**0.265**	**(<0.001)**	SAPS II	1.11 (1.04-1.17)	**0.001**
**Maximum lacate**	0.779	SAPS II	1.11 (1.04-1.18)	**0.001**	0.830	Vascular CM	2.87 (0.86-9.52)	0.086
	**(<0.001)**	Maximum lacate	1.70 (0.91-3.18)	0.096	**(<0.001)**	SAPS II	1.08 (1.01-1.15)	**0.003**
						Maximum lactate	2.29 (1.23-4.26)	**0.008**
**Mean lactate**	0.783	SAPS II	1.11 (1.04-1.18)	**0.001**	0.828	Vascular CM	2.83 (0.84-9.50)	0.093
	**(0.001)**	Mean lactate	2.45 (0.99-6.10)	0.055	**(<0.001)**	SAPS II	1.09 (1.02-1.17)	**0.008**
						Mean lactate	7.42 (1.61-34.20)	**0.010**
**Normalized lactate area score**	0.793	SAPS II	1.11 (1.05-1.18)	**0.001**	0.826	Vascular CM	2.81 (0.84-9.39)	0.093
	**(<0.001)**	Lactate area score	2.64 (1.08-6.49)	**0.034**	**(<0.001)**	SAPS II	1.09 (1.02-1.16)	**0.008**
						Lactate area score	7.04 (1.52-32.68)	**0.013**

Significant results are highlighted in bold. Abbreviations: AUROC=Area Under the Receiver Operating Characteristic Curve; OR=odds ratio; CI=confidence interval; CM=comorbidity; SAPS=Simplified Acute Physiology Score; SAH=S-Adenosylhomocysteine.
